# Influence of Obesity and Sociodemographic Features on the Physical Fitness of Breast Cancer Survivors

**DOI:** 10.3390/geriatrics9050125

**Published:** 2024-10-01

**Authors:** Malgorzata Biskup, Pawel Macek, Marek Zak, Halina Krol, Malgorzata Terek-Derszniak, Stanislaw Gozdz

**Affiliations:** 1 Collegium Medicum, Jan Kochanowski University, IX Centuries Kielc Avenue 19A, 25-516 Kielce, Poland; pawel.macek@onkol.kielce.pl (P.M.); mzak1@onet.eu (M.Z.); krolhalina@poczta.fm (H.K.); stanislaw.gozdz@onkol.kielce.pl (S.G.); 2 Department of Rehabilitation, Holycross Cancer Center, Artwinskiego 3 Street, 25-734 Kielce, Poland; m.terek@poczta.fm; 3 Department of Epidemiology and Cancer Control, Holycross Cancer Center, Artwinskiego 3 Street, 25-734 Kielce, Poland; 4 Department of Research and Education, Holycross Cancer Center, Artwinskiego 3 Street, 25-734 Kielce, Poland; 5 Department of Clinical Oncology, Holycross Cancer Center, Artwinskiego 3 Street, 25-734 Kielce, Poland

**Keywords:** breast cancer survivors (BCS), Senior Fitness Test (SFT), physical activity (PA), body mass index (BMI), waist circumference (WC), waist-to-hip ratio (WHR), waist-to-height ratio (WHtR), radiotherapy (RTH), chemotherapy (CHTH)

## Abstract

Introduction: Obesity is a chronic, relapsing, and progressive disease. The issue of obesity affects 50 to 80% of patients who have been diagnosed with breast cancer. The aim of this study is to assess the scale of the problem of obesity among breast cancer survivors (BCS) older than 60 years, evaluate their physical fitness, and study the relationship between the occurrence of obesity and levels of fitness among breast cancer survivors. The relationship between fitness and sociodemographic factors has also been analyzed. Materials and Methods: This original epidemiological clinical study included a cohort of 88 female breast cancer survivors treated in 2022 in Holycross Cancer Center, Kielce, Poland. A questionnaire involving demographic information and medical data was utilized. The Senior Fitness Test (SFT) battery has been used to assess the physical fitness of the patients. Body mass index (BMI), waist circumference (WC), waist-to-hip ratio (WHR), and waist-to-height ratio (WHtR) were also analyzed. Results: More than 70% of the studied breast cancer survivors were classified as overweight or obese, according to BMI and WC indices. Depending on the SFT trial, the regression coefficients indicated worse results in participants who were older, lived in urban areas, were professionally active, less educated, and had higher obesity indices. The analysis of the relationship between obesity indices and fitness components revealed that all obesity indices were significantly associated with lower body flexibility (BMI *p* = 0.0118, WC *p* = 0.0092, WHR *p* = 0.0364, WHtR *p* = 0.0095). Upper body flexibility was significantly correlated with BMI indices (*p* = 0.0091, *p* = 0.0193) and WHtR (*p* = 0.0095). Agility and balance were significantly associated with WC (*p* = 0.0193), WHR (*p* = 0.098), and WHtR (*p* = 0.0095). Lower body strength was significantly correlated with the WHR index (*p* = 0.0487). Significant differences were found in upper body strength depending on the WHtR category. Conclusions: In the studied group of breast cancer survivors, there is a high prevalence of overweight and obesity. With increasing obesity rates, older age, and lower education levels, physical activity measured by the SFT decreases. Living in urban areas and being professionally active also predispose to lower physical activity levels. As obesity indices increase, physical fitness decreases in trials assessing upper and lower body strength, upper and lower body flexibility, as well as agility and dynamic balance.

## 1. Introduction

Obesity not only elevates the risk of breast cancer and overall mortality but also increases the likelihood of developing a second cancer in breast cancer survivors (BCS) [1]. Defined as a chronic, progressive disease, obesity affects one-third of breast cancer patients at the time of their diagnosis. Additionally, 50% to 80% of patients gain further weight after their diagnosis [2]. It is estimated that 25% of global cancer cases are due to living a sedentary lifestyle as well as obesity. The International Agency for Research on Cancer has estimated that around 13,000 breast cancer cases could be prevented each year in the European Union by keeping a normal body weight [3,4,5]. The prevalence of obesity among breast cancer survivors is significantly higher than in the general population, with an increase of 3.0% per year in BCS compared to 2.3% in their cancer-free peers [2].

Due to being a complex problem, overweight and obesity are known critical global health challenges. They are complicated conditions, encompassing both the health aspect and also the socioeconomic domain. Before the 1990s, research into the causes of obesity was focused mainly on biological factors. Psychosocial factors were not explored enough. Sobal and Stunkard’s comprehensive review from the 1990s [6] has led to a new focus on the connections between socioeconomic status and the development of obesity. Currently, the prevalence of obesity is most often linked to unhealthy lifestyle choices, more than to genetic factors [7].

Cancer treatment impairs the activity of multiple systems, leading to reduced physical activity and, consequently, diminished physical performance and fitness [8]. Breast cancer treatment is a lengthy process and demands multiple intensive methods. Depending on the stage of the disease, surgery, chemotherapy (CHTH), radiotherapy (RTH), and hormonal therapy are used. In most cases, the treatment methods are used in combination, which, while increasing the success rate, also magnifies their adverse effects. Consequently, these treatments impair the locomotor, respiratory, nervous, and circulatory systems, leading to a decrease in fitness and aerobic endurance. These impairments manifest mostly as a decrease in the mobility of the joints of the pectoral girdle, the upper extremity, and spine, diminished muscle strength, lymphatic edema, and changes in posture. The mentioned changes, combined with the patients’ age, magnify the scale of the problem [9,10].

Assessment of physical fitness is essential both in research and practice. One of the standard tools for adults aged 60 or older is the Senior Fitness Test (SFT), introduced by Rikli and Jones in 1999 [11]. The results are typically interpreted using normative values, such as those proposed by Rikli and Jones [12]. Other authors publish different values for older adults in various regions, including Taiwan [13], Spain [14,15], Portugal [16], Hong-Kong [17], Chile [18] and Poland [19].

In the described group, physical fitness determines the quality of life, enabling the achievement of various goals, maintaining social contacts, mental health, and prevention of indolence and psychophysical disability [20].

Guidelines were published by the American Cancer Society, covering cancer survivors’ nutrition and physical activity. There are multiple ways that patients can receive support; therefore, it is crucial to provide patients with appropriate education. American Society of Clinical Oncology booklet “Managing Your Weight After a Cancer Diagnosis” (A Guide for Patients and Families, the American Cancer Society website or the National Comprehensive Cancer Network websites) are examples of valuable patient resources. In everyday practice, promoting undertaking and increasing physical activity could be an important factor in reducing body mass [4,21].

Understanding how physically active BCS are, what the scale of the problem of obesity is in this group, and how it influences overall fitness has crucial implications for public health. Regular physical activity and maintaining normal body mass allow older females to achieve a level of physical fitness that allows them to remain independent and live with dignity. At the same time, it is necessary to study the influence of sociodemographic factors (such as area of residence, education, or marital status) in order to direct increased attention, education, and exercise programs towards women in groups at higher risk of obesity and reduced fitness.

The study aims to assess the scale of the problem of obesity among BCS older than 60 years, as well as the relationship between obesity and fitness status among BCS. Additionally, we explored the interplay between fitness outcomes and sociodemographic characteristics.

## 2. Materials and Methods

### 2.1. Study Population

This original epidemiological clinical study included a group of 88 female breast cancer survivors from the Holycross Cancer Center in Kielce, Poland. The study was approved by the Ethics Committee in Kielce on 19 May 2017 (approval no. 19/2017). Participants were thoroughly briefed on the examination procedures and provided informed, written consent, a prerequisite for inclusion in the study. The entirety of the study was conducted in the Department of Rehabilitation of Holycross Cancer Center in 2022. All patients visiting the rehabilitation outpatient clinic were invited to participate in the study.

The following inclusion criteria were applied: a confirmed diagnosis of breast cancer, age above 60 years at the day of examination, the completion of surgical intervention (unilateral or bilateral mastectomy), female sex, a stable clinical state (adequate cardiovascular and respiratory function, not in a life-threatening condition, in good overall well-being), and the completion of RTH and/or CHTH. The exclusion criteria included male sex, terminal condition, blood pressure above 160/100 mmHg, and hospitalization on the day of assessment. Two patients were excluded from the study due to elevated blood pressure.

### 2.2. Physical Fitness Assessment

Physical fitness was assessed utilizing the SFT, which is the only test set recommended for seniors by the International Council of Sport Science and Physical Education. The test set was developed by Roberta Rikli and Jessie Jones from California State University in Fullerton, USA. Based on the research conducted by the authors, normal values for healthy elderly individuals were developed [22].

The SFT is a set of tests universally used for assessment of the physical fitness of older adults within a community context. It incorporates several tests, aimed at evaluating aerobic, neuro-motor, and musculoskeletal fitness [23], as well as overall health and intrinsic capacity [24,25,26]. The SFT consists of 6 trials, which assess fitness variables necessary to maintain independence and safe everyday activity. Each testing session begins with a 5–10-min warm-up and stretching exercises. Subjects are informed of the test procedures, and each trial is demonstrated before they attempt it. The patient is asked to perform to the best of their ability, without endangering their safety. Before and after each test, blood pressure and heart rate are measured.

The SFT consists of the following trials:

SFT1—30 s Chair Stand Test—assessment of the strength of the lower body.

The trial involves standing up and sitting down on a chair repeatedly. The subject is asked to perform possibly most repetitions of standing up. The number of repetitions is the result of the test. Reference values (min–max): 60–64 years 13–19; 65–69 years 12–18; 70–74 years 12–17; 75–79 years 11–17; 80–84 years 10–16; 85–89 years 10–15.

SFT2—30 s Arm Curl Test—assessment of the strength of the upper body.

The number of correctly performed flexions of the forearm in 30 s is the result of the study. Reference values (min–max): 60–64 years 12–17; 65–69 years 11–16; 70–74 years 10–15; 75–79 years 10–15; 80–84 years 9–14; 85–89 years 8–13.

SFT3—2-min Step-In-Place—assessment of aerobic endurance.

The trial involves raising knees to the height halfway between the patella and ilium. The number of correctly performed raises is the result of the trial. Reference values (min–max): 60–64 years 75—107; 65–69 years 73–107; 70–74 years 68–101; 75–79 years 68–100; 80–84 years 60–90; 85–89 years 55–85.

SFT4—Chair Sit-and-Reach Test—assessment of the lower body flexibility (in particular of the hamstrings).

The study participant sits on the edge of the chair. One of the participant’s lower extremities with the entire foot flat on the ground. The other, dominant extremity is extended, with the heel on the ground and the foot flexed at a right angle. The trial involves bending forward while maintaining the spine as straight as possible, with the head in line with the spine. The arms are extended forward, hands placed one on top of the other (middle fingers at the same level). The participant tries to touch the toes with their fingers. The result of the trial is the distance measured between the middle finger of the hand and the big toe. A positive (“+”) value indicates that the fingers surpassed the line of the toes, while a negative (“–“) one indicates that the fingers have not reached the line of the toes. Reference values (min–max): 60–64 years—−0.5–+5.0; 65–69 years –0.5–+4.5; 70–74 years −1.0–+4.0; 75–79 years −1.5–+3.5; 80–84 years −2.0–+3.0; 85–89 years −2.5–+2.5.

SFT5—Back Scratch Test—assessment of the upper body flexibility (in particular of the shoulders).

The study participant places one hand over the same shoulder, extending the fingers downward as far as possible. The other hand is placed behind the shoulder with the palm facing outward, stretching the fingers upward, trying to touch the fingers of both hands. The distance between middle fingers is the result of the trial. If the fingers overlap, the value is positive (“+”), if not—negative (“–“). Reference values (min–max): 60–64 years—−3.0–+1.5; 65–69 years −3.5–+1.5; 70–74 years −4.0–+1.0; 75–79 years −5.0–+0.5; 80–84 years −5.5–+0.0; 85–89 years −7.0–−1.0.

SFT6—8 Foot Up-and-Go Test—assessment of the patient’s agility and dynamic balance.

The trial starts with the patient sitting on a chair. The hands are placed on the knees, and the feet are flat on the ground. At the signal of the instructor, the participant stands up as fast as possible, using hands for support when necessary. They then move at maximum speed to a marker placed 2.44 m from the edge of the chair and return to the seated position. The test result is the time taken to complete the task, measured to the nearest tenth of a second. Reference values (min–max): 60–64 years—4.4–6.0; 65–69 years 4.8–6.4; 70–74 years 4.9–7.1; 75–79 years 5.2–7.4; 80–84 years 5.7–8.7; 85–89 years 6.2–9.6.

Utilization of the test allows for an assessment of physical fitness, understood as a set of skills. It enables a detailed analysis of these components and, therefore, proper training planning and effects control [22].

### 2.3. Demographics and Cancer Treatment Variables

In the study, a questionnaire was used to gather demographic details (such as age, educational background, marital status, and area of residence) and medical information, including treatment history, comorbidities, and occurrence of post-surgery lymphatic edema. Anthropometric measurements included body mass, height (self-reported by the participants), and waist (WC) and hip circumferences (measured by the personnel).

### 2.4. General Adiposity Assessment

The BMI (body mass index) for the individuals in the study was calculated using their anthropometric measurements: body mass in kilograms divided by the square of their height in meters [27,28,29,30].

### 2.5. Adipose Tissue Distribution Measurements

Waist and hip circumference measurements were obtained using a metric, nonstretchable tape measure level with the floor and noted with a precision of 0.1 cm, adhering to the guidelines for anthropometric measurements. Following the WHO protocol, WC was measured at the midpoint between the iliac crest and the lower margin of the last palpable rib, in the mid-axillary line. The widest point of the hips was used to measure hip circumference. A WC of less than 88 cm was considered normal, while a WC of 88 cm or more was indicative of central obesity. The waist-to-hip ratio (WHR) was computed by dividing the WC by the hip circumference, with values below 0.80 classified as normal and 0.80 or above indicating abdominal obesity. Additionally, the waist-to-height ratio (WHtR) was determined by dividing the WC by the person’s height [28,29,30,31].

### 2.6. Statistical Analysis

Baseline statistics are presented as means (with standard deviation), medians (with interquartile range), ranges (minimum–maximum), or numbers/proportions, depending on the nature of the studied variable. The distribution of continuous variables was assessed utilizing the Shapiro–Wilk test. The results of this testing implied the need to use nonparametric tests. Statistical differences in SFT results, depending on the analyzed categories of sociodemographic features and values, were tested using the Mann–Whitney test. Effects sizes were demonstrated using the rank-biserial correlation. Relationships of specific SFT trials (based on the norms established by Roberta Rikli and Jessie Jones [22]) with clinical and sociodemographic, as well as obesity status, were analyzed using the chi-square test. The relationship of results received in the individual SFT trials (continuous variables), sociodemographic features, and obesity status were analyzed utilizing robust regression models. Both single- and multi-factor models were fitted. Multi-factor models for each SFT trial were created based on backward stepwise regression. The full model incorporates all the studied independent variables. For each variable, statistical significance was assessed. Whenever the *p*-value for a variable was higher than the fixed level of significance, the variable was removed from the model. *p*-values of less than 0.05 are considered statistically significant. All statistical analyses were performed using R software (ver. 3.6.3).

## 3. Results

### 3.1. Basic Characteristics of the Studied Group

The study included 88 female breast cancer survivors. The average age was 69.2 ± 5.9 years. The average time from diagnosis to study enrollment was 9.2 ± 7.9 months. Adjuvant RTH and/or CHTH were used equally, in approximately half of the patients (Table 1). Seventy-five percent of the patients resided in urban areas; more than half reported being in a relationship. Eighty-one percent of the patients had higher education. An absence of professional activity and the presence of comorbidities were observed in 95% and 86% of the patients, respectively. More than 70% of the studied females were classified as overweight or obese, according to BMI and WC indices. Values of WHR and WHtR indicated obesity in more than 90% of the studied group.

### 3.2. Senior Fitness Test

Significant differences in the SFT3 trial were found in correlation with the level of education; for SFT4, with the area of residence and level of education; for SFT5 (right hand), with age; and for SFT6, with marital and occupational status (Tables S1 and S2—Supplementary Materials). The differences in the results of SFT1 and SFT4 in correlation with occupational status, and the results of SFT5 (left hand) in correlation with the patient’s age, were at the threshold of statistical significance (Table 2).

Significant differences were observed in the results of SFT1, SFT5 (right hand), and SFT5 (left hand) depending on the BMI. For SFT4, SFT5 (right hand), SFT5 (left hand), and SFT6, these differences were associated with waist circumference (WC). Additionally, significant variations were noted in SFT1, SFT2, SFT3, SFT5 (right hand), and SFT5 (left hand) depending on the WHtR (Tables S2 and S3—Supplementary Materials). Notably, the differences in SFT1 associated with WC, and in SFT3 associated with WHtR, were at the threshold of statistical significance.

The relationship between clinical, sociodemographic features, and obesity categories with specific SFT trial results (categorized as “below normal” and “normal” to “above normal”) has been studied (Table S4—Supplementary Materials). The relationship between SFT2 results and lymphadenectomy status was statistically significant (Table S4—Supplementary Materials). The patients who underwent axillary lymphadenectomy were more likely to score lower in this trial. The relationship between SFT1, SFT3, and SFT5 (right hand) with the level of education was statistically significant as well, with higher-educated patients more likely to be in the “normal” to “above normal” range. Occupational status was significantly related to SFT4, SFT5 (right hand), and SFT6 results. In these trials, the professionally inactive individuals were more likely to score “below normal”. The relationship of obesity indices: BMI with SFT5 (right hand), SFT5 (left hand); WC with SFT4, SFT5 (right hand), SFT5 (left hand), SFT6, as well as WHtR with SFT4, SFT5 (right hand), and SFT5 (left hand) were statistically significant. Patients BMI and WC values suggesting overweight or obesity were more likely to achieve results “below normal”.

In the univariate regression models, presented in Table 3, negative regression coefficients indicate a relative reduction, while positive values suggest a relative increase in the results of SFT trials (excluding SFT6). In SFT6, lower measurement values correspond to better scores. SFT1 results were significantly related to age, occupational status, and WtHR. SFT results were significantly related to the level of education; SFT4 correlated with educational and occupational status, BMI, WC, WHR, and WHtR. SFT6 results were related to age, area of residence, occupational status, BMI, WHR, and WHtR. SFT5 (left hand) results were related to BMI, WC, and WHtR. The relationship between SFT2 and occupational status, SFT3 and age, SFT4 and area of residence, as well as SFT6 and marital status were on the threshold of statistical significance. Depending on the SFT trial, the regression coefficients pointed to worse results in the elderly, patients living in urban areas, with lower education, professionally active, and with higher obesity indices, except for WHtR in SFT6.

Multivariate analyses included (depending on the presented model): age, area of residence, marital status, level of education, occupational status, comorbidities, BMI, WC, WHR, WHtR, and time from breast cancer diagnosis to the date of study enrollment. According to the results from the multivariate regression models (Table 4), the results of SFT1 were related to age, level of education, and occupational status; SFT3 results were related to age, level of education, and time from diagnosis to enrollment; SFT4 showed a significant relationship with the level of education and occupational status; SFT5 (right hand) was related to occupational status and WHtR; and SFT6 was related to age, WC, and time from the diagnosis to enrollment. For SFT2 and SFT5 (left hand), multivariate regression models could not be fitted.

## 4. Discussion

Epidemiological data [7] indicates a continuous increase in breast cancer incidence, with treatment often leading to limitations of physical activity [32]. Therefore, it is reasonable to conduct research on the fitness of elderly females undergoing treatment for breast cancer.

Research [33,34] comparing elderly breast cancer survivors to healthy individuals confirms that survivors report worse physical conditions than their cancer-free peers. Our research supports this finding, demonstrating that females treated for breast cancer are significantly less functionally fit compared to the normal values developed for healthy individuals. The largest limitations were observed in the upper body flexibility test (SFT5), which examines the patient’s range of movement, as well as in the agility and dynamic balance test (SFT6). The literature [35,36,37] confirms the limitation of the range of movement in patients who have undergone breast cancer surgery, despite the surgery not damaging the tissues surrounding the shoulder joint. A 2019 review encompassing 1032 studies found that 1–67% of all patients reported limitations in shoulder movements, 0–34% reported lymphatic edema, 9–68% reported shoulder/arm pain, and 9–28% reported arm weakness [36]. According to McNeely, the problems with shoulder movement are reported by 42–56% of patients, and 10–55% of patients demonstrate decreased range of movement of the glenohumeral joint [38]. The discrepancies between studies arise from differences in research methodologies. The development of scar tissue as well as nerve damage resulting from surgery are potential causes of this limitation [39,40,41,42,43]. The problem can also arise due to post-CHTH or post-RTH tissue fibrosis [37,44]. Over time, some patients experience a varying degree of improvement of the shoulder joint mobility, often as pain subsides and mobility in daily activities increases. However, there are types of movement that can exacerbate limitations. Contractures of juxta articular soft tissue and hardening of the scar contribute to this phenomenon [45]. The limitation of joint movement and weakening of muscle strength can lead to the development of postural abnormalities and disorders of static balance [46]. Comparing mobility across various studies is challenging due to variance in utilized measurement methods and the time elapsed since surgery. The study included patients who underwent their treatment on average 9 years prior. It is therefore inappropriate to refer to studies describing patients who are 6 months [47], 18 months [48], 32 months [49], 44 months [50], 47 months [51], or 51 months [52] post-treatment.

A decrease in mobility in females treated for breast cancer is also evident in the long term. A study by Hidding et al. demonstrated a 10% decrease in flexion more than 5 years post-treatment [53]. Another study found a loss of motion of 20 degrees or more in patients 7 years post-surgery [39]. In a study by Fisher et al., among breast cancer patients, most of the range of motion measurements were decreased by 4–12% even 4 years after completion of treatment, compared to their healthy counterparts [52]. This research demonstrates a decrease in upper extremity flexibility with increasing age. Other studies [54] assessing flexibility, in particular of the upper body, confirm this finding.

The upper extremity flexibility test was performed actively by our study participants. However, not all studies provide information on the measurement process and whether the range of motion is measured actively or passively [49,55,56].

The investigation does not confirm the detrimental effect of RTH on upper extremity mobility, which may be attributed to the average time of 9 years from surgery. During this period, many patients underwent physiotherapeutic interventions. The correlation between clinical data and radiation dosage regarding shoulder and arm damage has been previously explored [36,50].

In this analysis, we also assessed both upper and lower extremities’ strength using the forearm flexion and chair stand trials—components of the SFT. We found that 19 exhibited below-normal upper limb strength, and 23 had below-normal lower limb strength. Muscle strength is determined by several factors, many of which are individual in nature, such as muscle structure, stimulus strength, frequency of stimulation, and body mass of the studied person. It is also influenced by circadian rhythm and occupational workload [57]. We hypothesize that decreased strength in the studied group was associated with an energy-conserving lifestyle caused by fear of developing lymphatic edema. In the study, the patients who underwent lymphadenectomy were significantly more likely to have below-normal results of the upper extremity strength test. The variability in human anatomy complicates the definition of lymphatic edema, as. not every “healthy” individual has an equal upper extremity circumference, making it challenging to set a clear threshold for the occurrence of swelling [58]. Both strength and aerobic endurance deteriorate with age [54], which is confirmed by our findings. The deterioration is often attributed to sarcopenia—the age-related loss of muscle mass, which leads to reduced skeletal muscle strength. Consequently, decreasing levels of physical activity lead to a decrease in muscle mass and aerobic endurance [54]. A decrease in upper extremity strength of 10% to 15% is commonly reported 1–5 years after breast cancer treatment. Aerobic endurance and ability to sustain activity over time among female breast cancer patients have been less extensively studied, with results varying from no deficit to a 20% aerobic endurance deficit [52]. Rasmussen et al. highlight that body size should be taken into account while assessing muscle strength, as it is a known confounding factor in strength tests [37].

We demonstrated values lower than the recommended levels in the SFT6 trial, which assesses gait and balance. Nawara [59] discovered a correlation between the participants’ age and the SFT6 trial results. These findings are consistent with the studies conducted by Cadore et al. [60], which indicate that balance and gait speed decline with age. However, our study did not confirm this relationship, which could result from the small sample size.

We also assessed sociodemographic factors. Depending on the SFT trial, the regression coefficients pointed to worse results in the elderly, people living in urban areas, with lower education status, and those who are professionally active. A nationwide study on 7000 United States citizens aged 60–94 assessed using SFT confirms that the results of all fitness tests results deteriorate with age, declining by 10–15% a decade [11]. Unfortunately, a lack of literature on breast cancer survivors in this domain precludes a direct comparison with findings from other researchers.

The results of our study demonstrate statistically significant differences in the body mass of breast cancer survivors. Overweight and obesity measured using BMI and WC were found in around 70% of participants. WHR and WHtR values pointed to obesity in more than 90% of the studied females. Depending on the SFT trial, the regression coefficients showcased worse results of these trials in females with higher obesity indices, except for WHtR in the SFT6 trial. A general trend observed is that with age, individuals tend to lose height and increase body mass until the age of 70, after which body mass tends to decrease [61]. There is a consensus among researchers that using BMI, which relies on height measurements, may not be the most accurate method for assessing body composition in the elderly. In this group, there is often an increase in thoracic kyphosis and flattening of intervertebral discs, which significantly reduces a person’s height, potentially leading to an overestimation of the BMI [62]. This can result in classifying underweight individuals as having normal body weight and ones with normal body structure as overweight. Yilmaz et al. propose arm length as a substitute for height when calculating BMI in the elderly [55,63]. Our research also assesses physical fitness in relation to BMI and adipose tissue distribution, demonstrating lower fitness in lower and higher extremity flexibility tests, lower extremity strength tests, as well as agility and dynamic stability tests. Research papers [64,65,66] confirm a decrease in physical fitness as body mass increases. The elderly population is often characterized by an increased concentration of adipose tissue and a decrease in muscle mass and bone density, compared to younger adults. A recent study [67] showed that obesity in the elderly tends to be localized around the abdominal area, combined with a decrease in subcutaneous adipose tissue as they age. Abdominal obesity is also correlated with various types of cancer [68,69] and has been reported as a risk factor for disability in the elderly [70]. Barbosa et al. [71], in a 4-year observational study, highlighted that abdominal obesity significantly increases the risk of motor disability in patients aged 64–74 years. Moreover, abdominal obesity with accompanying dynapenia (also known as dynapenic abdominal obesity) significantly increases the risk of falls among the elderly [72]. A review by Chen et al. consolidates these findings, concluding that abdominal obesity in the elderly threatens their abilities to walk, climb stairs, and rise from chairs, subsequently reducing their quality of life [73]. In our research, normal WC was found only in 21.1% of participants. High WC in the studied group corresponds to abdominal obesity. The studied females were in the menopausal age, when a decrease in circulating estrogens causes an alteration in the adipose tissue biology, and leads to a redistribution of adipose tissue mass, moving it to the abdominal area, as observed in breast cancer patients [46]. Our study aimed to investigate the associations between fitness test outcomes and prevalence of abdominal obesity among older breast cancer survivors. We demonstrated the relationship between abdominal obesity and function tests in the upper and lower extremity flexibility tests, as well as the agility and dynamic stability tests. Chen et al. confirm the relationship between physical activity and abdominal obesity [24]. Other authors also highlight the negative influence of abdominal obesity on maintaining body balance [24,74]. Dulac et al. [75] observed a correlation between WC and the results of the chair stand test (lower extremity strength test) among older females. However, their study demonstrated that fitness may not be related to abdominal obesity but rather to lower extremity muscle strength. While aging is inherently linked to decreased physical fitness [76,77], increasing muscle strength can slow this process down and help to maintain functional skills in the elderly. The measurements of fitness are more related to muscle strength [78]. In order to maintain the ability to live independently, the elderly should focus on muscle strength to avoid sarcopenia rather than focusing solely on maintaining a slim figure [24,79]. Vincent et al. [73] highlight that aging is related to progressive muscle mass loss and an increase in adipose tissue. Cespedes [80] notes that more than one-half of older patients with cancer are frail or prefrail at cancer diagnosis. In addition to screening for frailty in oncology settings, exercise and physical activity interventions may be beneficial in this setting. In the general population, physical activity interventions (e.g., resistance and aerobic exercise, brisk walking, or balance training) help to mitigate physical frailty and maintain independence in older adults. Nutrition support may also play a role, although results of multimodal interventions combining nutrition and physical activity have been mixed. Among cancer survivors, physical activity interventions are effective in preserving or increasing physical function.

The study group presents complex assessment challenges as age-related changes coexist with adverse events caused by oncological treatment, including changes in the musculoskeletal system [9,10]. The differences in adipose tissue distribution can impair functional fitness in various ways. In this study, we employed various methods to assess obesity. Although the studied sample was small, the presented observations can motivate the control of body mass and WC in female breast cancer survivors. Regular examination of chosen body mass parameters can be helpful while controlling the process of post-mastectomy rehabilitation. Interventions aimed at limiting the increase in body mass after breast cancer diagnosis can significantly improve physical fitness and survival [46].

## 5. Strengths and Weaknesses of the Study

Our study has several limitations. It is based on a relatively small study group and is not an international research project. The studied females all live in Europe (Poland), while the physical activity has been assessed utilizing the normal values developed for the United States population. Future plans for this project include expanding the participant pool to incorporate a control group of females without breast cancer. It is also recommended to perform fitness tests at various stages of oncological treatment, as well as before its start, to enhance the reliability of the data. There was no control group of patients without breast cancer, the inclusion of which could have further enhanced the quality of the analysis. Another limitation is that our sample was not systematically chosen for the protocol project but was instead randomly chosen during control visits, leading to inconsistencies in the types of surgery undergone (breast conserving therapy, mastectomy). The next factor that could have impacted our results was that dietary records were not collected.

The SFT is designed so that everyone who can move unaided is able to perform it. However, there are some limitations in its utilization. The test in its full form cannot be used in patients with severe damage to the motor or balance systems, in whom safety considerations preclude participation in trials requiring movement. The limitations of this cross-sectional study include sampling only older adults who are independent, meaning the results cannot be extrapolated to older individuals with decreased physical fitness. It should be considered to collect long-term data from individuals with varying physical and mental conditions to broaden the applicability of the findings.

Nevertheless, this study possesses multiple strengths. The SFT is a reliable and important test for assessing functional fitness. It facilitates the evaluation of physical fitness levels through simple motor tasks that are quick to administer and easy to interpret. The ease of interpretation is vital for effective patient communication, which is a crucial element of rehabilitation. The use of simple, uncomplicated motor patterns derived from everyday life allows for an indirect assessment of physical fitness parameters, such as strength and elasticity of the upper and lower body, aerobic endurance, motor coordination, and reaction time. The test set is safe for the elderly and easy to perform, requiring no complicated devices or specialized facilities [54].

Several studies have utilized the SFT to assess the fitness of the elderly, including analyses of healthy individuals [54] and nursing home residents [61]. Fitness levels are analyzed depending on the completed training programs [74].

Our results are in agreement with findings by other researchers [81,82], who have noted that screening tools and the Comprehensive Geriatric Assessment in geriatrics are instrumental in identifying patients who are most susceptible to suboptimal outcomes from cancer treatments and helpful in the customization of therapeutic approaches. Implementing various evidence-based strategies within geriatric care can enhance overall conditions for the elderly, subsequently improving interactions between clinicians, patients, and caregivers, and boosting clinical outcomes for this demographic.

## 6. Conclusions

In the studied group of breast cancer survivors, there is a high prevalence of overweight and obesity. More than 70% of the studied females were classified as overweight or obese, according to BMI and WC indices. Values of WHR and WHtR indicated obesity in more than 90% of the studied group. Physical fitness of older breast cancer survivors falls below recommended norms. The most limited domains are flexibility of the upper extremity, as well as agility and balance.

Social factors significantly influence the levels of physical fitness among breast cancer survivors. With increasing obesity rates, older age, and lower education levels, physical activity measured by the SFT decreases. Living in urban areas and being professionally active also predispose to lower physical activity levels.

Flexibility of the upper body, strength of the lower body, strength of the lower body, agility, and dynamic balance all decrease with age. With an increase in the level of education, we observe an increase in lower extremity strength, strength of the lower body, aerobic endurance, and upper and lower body flexibility. The professionally inactive BCS scored less in tests related to strength and flexibility of the lower body, upper body flexibility, agility, and dynamic balance. BCS residing in urban areas demonstrated reduced body flexibility, agility, and dynamic balance.

As obesity indices increase, physical fitness decreases in trials assessing upper and lower body strength, upper and lower body flexibility, as well as agility and dynamic balance.

## 7. Implications for Clinical Practice

Elderly female patients comprise a large part of the breast cancer patient population, yet not much is known about the effects and consequences of breast cancer treatment in this age group. Our study demonstrates that in the studied group, the prevalence of overweight and obesity poses a significant challenge. Older patients do not regain physical fitness after undergoing surgery and adjuvant treatments for breast cancer. For the elderly, the influence of treatment on physical fitness and independence can be more crucial than the direct treatment outcomes themselves. Both clinicians and elderly patients should be aware of the influence of treatment on physical functioning. We should prevent the deterioration of physical fitness in the elderly breast cancer survivors to avoid loss of independence.

The observations presented should encourage increased control over body mass and WC in female BCS. Routine follow-up of selected body mass indices and physical fitness should be standard in the post-mastectomy group. Interventions aimed at limiting increases in body mass after the diagnosis can significantly enhance physical fitness.

## Figures and Tables

**Table 1:** Basic characteristics of the studied group (n = 88; 100%).

Characteristic	n (%)
Age (year)	
Mean (SD)	69.2 (5.9)
Median (IQR)	68.0 (8.0)
Min–max	60.0–85.0
Age group	
60–64 years	20 (22.7)
65–69 years	35 (39.8)
70–74 years	14 (15.9)
75 or older	19 (21.6)
Mastectomy side	
Both sides	11 (12.5)
Left side	47 (53.4)
Right side	30 (34.1)
Lymphadenectomy	
No	56 (63.6)
Yes	32 (36.4)
RTH	
No	46 (52.3)
Yes	42 (47.7)
CHTH	
No	44 (50.0)
Yes	44 (50.0)
Area of residence	
Rural	22 (25.0)
Urban	66 (75.0)
Marital status	
In a relationship	51 (58.0)
Single	37 (42.1)
Education	
Higher level	72 (81.8)
Lower level	16 (18.2)
Occupational status	
Professionally active	4 (4.6)
Professionally inactive	84 (95.5)
Comorbidities	
No	12 (13.6)
Yes	76 (86.4)
BMI category	
Normal weight	22 (25.0)
Overweight or obesity	66 (75.0)
WC category	
Normal	23 (26.1)
Abdominal obesity	65 (73.9)
WHR category	
Normal	5 (5.7)
Abdominal obesity	83 (94.3)
WHtR category	
Normal	7 (7.9)
Obesity	81 (92.1)
Time from diagnosis to study enrollment (years)	
Mean (SD)	9.2 (7.9)
Median (IQR)	7.0 (11.0)
Min–max	1.0–41.0

Note: Data presented as number (percentage), unless stated otherwise. Abbreviations: RTH, radiotherapy; CHTH, chemotherapy; BMI, body mass index; WC, waist circumference; WHR, waist-to-hip ratio; WHtR, waist-to-height ratio; SD, standard deviations; IQR, interquartile range; min–max, minimum–maximum.

**Table 2:** Basic characteristics of the SFT results according to the categories of sociodemographic features.

Characteristic		30 s Chair Stand Test	30 s Arm Curl Test	2 min Step-In-Place	Chair Sit-and-Reach Test	Back Scratch Test (Right Hand)	Back Scratch Test (Left Hand)	8 Foot Up-and-Go Test
Age group								
60–64 years	Mean (SD)	12.70 (3.01)	16.75 (8.05)	86.90 (15.22)	−4.80 (7.84)	−11.05 (12.36)	−15.95 (11.91)	8.55 (1.96)
	Median (IQR)	12.50 (5.00)	16.00 (7.50)	88.50 (18.50)	−3.00 (8.00)	−8.00 (20.50)	−13.00 (18.00)	8.50 (1.50)
	Min–max	7.00–17.00	8.00–46.00	50.00–110.00	−21.00–6.00	−36.00–5.00	−36.00–5.00	5.00–13.00
65–69 years	Mean (SD)	11.91 (2.72)	15.40 (4.22)	81.09 (28.58)	−4.09 (9.08)	−3.91 (8.51)	−10.09 (10.74)	9.17 (2.68)
	Median (IQR)	12.00 (2.00)	15.00 (5.00)	75.00 (47.00)	0.00 (12.00)	0.00 (14.00)	−9.00 (14.00)	9.00 (1.00)
	Min–max	5.00–19.00	3.00–23.00	26.00–133.00	−33.00–8.00	−25.00–7.00	−39.00–5.00	6.00–19.00
70–74 years	Mean (SD)	11.57 (2.38)	16.36 (5.30)	75.79 (21.21)	−6.07 (9.73)	−13.43 (13.61)	−17.00 (13.99)	9.57 (3.25)
	Median (IQR)	12.00 (2.00)	18.00 (10.00)	75.00 (18.00)	−1.00 (16.00)	−14.00 (23.00)	−20.50 (22.00)	9.00 (6.00)
	Min–max	6.00–15.00	8.00–23.00	42.00–126.00	−25.00–4.00	−38.00–6.00	−37.00–7.00	6.00–17.00
75 or older	Mean (SD)	10.89 (4.36)	15.05 (3.54)	73.42 (29.30)	−6.21 (10.60)	−10.58 (18.06)	−16.16 (17.53)	12.53 (6.35)
	Median (IQR)	11.00 (6.00)	16.00 (4.00)	65.00 (50.00)	−3.00 (16.00)	−9.00 (20.00)	−18.00 (15.00)	11.00 (10.00)
	Min–max	1.00–20.00	8.00–24.00	43.00–127.00	−29.00–10.00	−64.00–23.00	−39.00–40.00	5.00–25.00
Area of residence								
Rural	Mean (SD)	11.77 (3.02)	16.55 (7.58)	73.55 (21.81)	−8.45 (10.11)	−11.18 (11.85)	−17.64 (11.89)	8.77 (1.82)
	Median (IQR)	12.00 (4.00)	15.00 (7.00)	71.00 (33.00)	−5.50 (18.00)	−10.00 (19.00)	−16.50 (20.00)	9.00 (2.00)
	Min–max	7.00–17.00	8.00–46.00	26.00–104.00	−33.00–4.00	−36.00–6.00	−36.00–5.00	5.00–13.00
Urban	Mean (SD)	11.83 (3.23)	15.53 (4.37)	82.03 (26.13)	−3.88 (8.59)	−7.59 (13.46)	−12.56 (13.66)	10.17 (4.41)
	Median (IQR)	12.00 (3.00)	16.00 (5.00)	77.50 (41.00)	0.00 (11.00)	−5.00 (17.00)	−11.50 (16.00)	9.00 (3.00)
	Min–max	1.00–20.00	3.00–24.00	39.00–133.00	−29.00–10.00	−64.00–23.00	−39.00–40.00	5.00–25.00
Marital status								
In a relationship	Mean (SD)	12.02 (2.89)	16.20 (5.78)	78.88 (23.95)	−4.65 (8.52)	−9.51 (13.30)	−14.71 (12.77)	9.10 (3.43)
	Median (IQR)	12.00 (4.00)	15.00 (7.00)	77.00 (35.00)	−2.00 (10.00)	−9.00 (19.00)	−14.00 (19.00)	8.00 (1.00)
	Min–max	6.00–19.00	8.00–46.00	39.00–133.00	−24.00–10.00	−64.00–7.00	−39.00–5.00	5.00–24.00
Single	Mean (SD)	11.54 (3.53)	15.22 (4.65)	81.32 (27.26)	−5.54 (10.06)	−7.08 (12.87)	−12.62 (14.22)	10.81 (4.46)
	Median (IQR)	12.00 (3.00)	16.00 (7.00)	76.00 (41.00)	−3.00 (10.00)	−5.00 (16.00)	−12.00 (13.00)	9.00 (4.00)
	Min–max	1.00–20.00	3.00–24.00	26.00–127.00	−33.00–6.00	−36.00–23.00	−37.00–40.00	5.00–25.00
Education								
Higher level	Mean (SD)	12.03 (3.19)	15.92 (5.63)	83.36 (24.50)	−3.76 (8.32)	−7.63 (13.14)	−12.85 (13.06)	9.94 (4.28)
	Median (IQR)	12.00 (3.00)	15.50 (5.50)	78.00 (35.50)	0.00 (9.50)	−5.00 (18.50)	−12.00 (14.00)	9.00 (2.00)
	Min–max	1.00–20.00	3.00–46.00	42.00–133.00	−29.00–10.00	−64.00–23.00	−39.00–40.00	5.00–25.00
Lower level	Mean (SD)	10.88 (2.94)	15.19 (3.71)	64.38 (23.39)	−10.69 (10.81)	−12.38 (12.59)	−18.25 (14.20)	9.25 (2.05)
	Median (IQR)	10.50 (5.50)	15.50 (7.00)	59.00 (31.00)	−9.00 (19.00)	−10.00 (13.00)	−21.00 (26.50)	9.00 (2.00)
	Min–max	7.00–16.00	10.00–20.00	26.00–108.00	−33.00–4.00	−36.00–5.00	−38.00–5.00	6.00–13.00
Occupational status								
Professionally active	Mean (SD)	14.25 (1.71)	18.75 (2.99)	93.50 (15.78)	2.50 (3.00)	0.75 (1.89)	−10.25 (15.44)	6.00 (1.41)
	Median (IQR)	14.50 (2.50)	19.00 (4.50)	96.00 (19.00)	2.00 (5.00)	1.50 (2.50)	−7.00 (24.50)	5.50 (2.00)
	Min–max	12.00–16.00	15.00–22.00	72.00–110.00	0.00–6.00	−2.00–2.00	−30.00–3.00	5.00–8.00
Professionally inactive	Mean (SD)	11.70 (3.18)	15.64 (5.38)	79.26 (25.51)	−5.38 (9.20)	−8.93 (13.25)	−14.00 (13.33)	10.00 (3.96)
	Median (IQR)	12.00 (3.00)	15.00 (6.00)	76.00 (35.00)	−2.50 (10.00)	−7.50 (19.00)	−12.00 (16.00)	9.00 (2.00)
	Min–max	1.00–20.00	3.00–46.00	26.00–133.00	−33.00–10.00	−64.00–23.00	−39.00–40.00	5.00–25.00
Comorbidities								
No	Mean (SD)	11.75 (2.18)	16.83 (3.79)	79.33 (14.34)	−4.25 (9.40)	−8.67 (12.48)	−16.33 (8.86)	9.92 (5.09)
	Median (IQR)	11.50 (3.00)	16.50 (5.00)	77.00 (16.50)	0.00 (8.00)	−7.00 (15.50)	−15.00 (14.00)	8.50 (1.50)
	Min–max	9.00–16.00	10.00–23.00	62.00–110.00	−25.00–6.00	−36.00–6.00	−33.00–3.00	6.00–25.00
Yes	Mean (SD)	11.83 (3.30)	15.62 (5.53)	80.00 (26.65)	−5.14 (9.17)	−8.46 (13.28)	−13.43 (13.94)	9.80 (3.80)
	Median (IQR)	12.00 (4.00)	15.00 (6.00)	76.50 (37.50)	−2.00 (10.50)	−6.50 (18.50)	−12.00 (16.50)	9.00 (2.00)
	Min–max	1.00–20.00	3.00–46.00	26.00–133.00	−33.00–10.00	−64.00–23.00	−39.00–40.00	5.00–24.00

Abbreviations: 30 s Chair Stand Test, 30 s Arm Curl Test, 2 min Step-In-Place, Chair Sit-and-Reach Test, Back Scratch Test (right hand) Back Scratch Test (left hand) and 8 Foot Up-and-Go Test, specific tests included in the SFT; SD, standard deviations; IQR, interquartile range; min–max, minimum–maximum.

**Table 3:** Associations of sociodemographic features with SFT results based on univariate regression models.

Characteristic	30 s Chair Stand Test		30 s Arm Curl Test		2 min Step-In-Place		Chair Sit-and-Reach Test		Back Scratch Test (Right Hand)		Back Scratch Test (Left Hand)		8 Foot Up-and-Go Test	
	Estimate (95% CI)	*p*	Estimate (95% CI)	*p*	Estimate (95% CI)	*p*	Estimate (95% CI)	*p*	Estimate (95% CI)	*p*	Estimate (95% CI)	*p*	Estimate (95% CI)	*p*
Age (year)	−0.14 (−0.27, −0.01)	0.0341	−0.07 (−0.24, 0.10)	0.4349	−0.88 (−1.84, 0.07)	0.0688	−0.12 (−0.45, 0.20)	0.4464	−0.06 (−0.64, 0.52)	0.8443	0.02 (−0.69, 0.72)	0.9643	0.29 (0.10, 0.49)	0.0029
Area of residence														
Urban vs. rural	0.06 (−1.44, 1.57)	0.9364	−1.02 (−4.40, 2.37)	0.5529	8.48 (−2.75, 19.72)	0.1371	4.58 (−0.20, 9.35)	0.0601	3.59 (−2.42, 9.60)	0.2379	5.08 (−0.97, 11.12)	0.0989	1.39 (0.07, 2.72)	0.0399
Marital status														
Single vs. in a relationship	−0.48 (−1.89, 0.93)	0.5002	−0.98 (−3.19, 1.23)	0.3810	2.44 (−8.68, 13.57)	0.6637	−0.89 (−4.95, 3.16)	0.6625	2.43 (−3.17, 8.03)	0.3913	2.08 (−3.77, 7.93)	0.4807	1.71 (−0.03, 3.46)	0.0539
Education														
Lower vs. higher	−1.15 (−2.79, 0.49)	0.1664	−0.73 (−3.00, 1.54)	0.5243	−18.99 (−31.95, −6.02)	0.0046	−6.92 (−12.64, −1.21)	0.0182	−4.75 (−11.72, 2.22)	0.1791	−5.40 (−13.10, 2.29)	0.1662	−0.69 (−2.12, 0.73)	0.3366
Occupational status														
Professionally active vs. professionally inactive	−2.55 (−4.38, −0.72)	0.0070	−3.11 (−6.30, 0.08)	0.0561	−14.24 (−30.87, 2.39)	0.0924	−7.88 (−11.47, −4.29)	0.0000	−9.68 (−13.11, −6.24)	0.0000	−3.75 (−19.36, 11.86)	0.6342	4.00 (2.35, 5.65)	0.0000
ComorbiditiesYes														
Yes vs. no	0.08 (−1.38, 1.54)	0.9147	−1.21 (−3.73, 1.30)	0.3390	0.67 (−9.56, 10.90)	0.8972	−0.89 (−6.68, 4.89)	0.7593	0.21 (−7.57, 7.98)	0.9581	2.90 (−3.09, 8.89)	0.3390	−0.11 (−3.16, 2.93)	0.9409
BMI (kg/m^2^)	−0.07 (−0.21, 0.08)	0.3702	−0.09 (−0.26, 0.08)	0.3073	−0.04 (−1.14, 1.07)	0.9466	−0.46 (−0.82, −0.10)	0.0118	−0.59 (−1.03, −0.15)	0.0091	−0.62 (−1.09, −0.15)	0.0103	0.03 (−0.09, 0.16)	0.5851
WC (cm)	−0.04 (−0.09, 0.01)	0.1516	−0.06 (−0.12, 0.01)	0.0780	0.05 (−0.35, 0.45)	0.8022	−0.16 (−0.28, −0.04)	0.0092	−0.19 (−0.39, 0.01)	0.0642	−0.23 (−0.44, −0.01)	0.0388	0.08 (0.01, 0.14)	0.0193
WHR	−10.90 (−21.73, −0.06)	0.0487	−14.47 (−30.20, 1.26)	0.0708	−19.12 (−107.34, 69.10)	0.6676	−32.49 (−62.88, −2.10)	0.0364	−21.68 (−69.21, 25.84)	0.3669	−27.84 (−65.23, 9.54)	0.1424	18.64 (4.60, 32.67)	0.0098
WHtR	−6.82 (−14.52, 0.87)	0.0814	−8.50 (−18.06, 1.05)	0.0805	−0.57 (−63.27, 62.12)	0.9855	−25.03 (−43.79, −6.27)	0.0095	−25.03 (−43.79, −6.27)	0.0095	−25.03 (−43.79, −6.27)	0.0095	−25.03 (−43.79, −6.27)	0.0095

Abbreviations: 30 s Chair Stand Test, 30 s Arm Curl Test, 2 min Step-In-Place, Chair Sit-and-Reach Test, Back Scratch Test (right hand) Back Scratch Test (left hand) and 8 Foot Up-and-Go Test, specific tests included in the SFT; BMI, body mass index; WC, waist circumference; WHR, waist-to-hip ratio; WHtR, waist-to-height ratio; 95% CI, 95% confidence interval.

**Table 4:** Associations of sociodemographic features with Senior Fitness Test results based on multivariate regression models.

Senior Fitness Test	Model	Estimate (95% CI)	*p*
30 s Chair Stand Test	Age	−0.16 (−0.29, −0.03)	0.0138
	Education—lower vs. higher level	−1.72 (−3.33, −0.11)	0.0365
	Occupational status—professionally inactive vs. professionally active	−1.71 (−3.37, −0.05)	0.0439
30 s Arm Curl Test	WHR	−14.47 (−30.20, 1.26)	0.0708
2 min Step-In-Place	Age	−2.01 (−2.76, −1.27)	0.0000
	Education—lower vs. higher level	−24.49 (−35.98, −13.00)	0.0001
	Follow-up	1.31 (0.74, 1.89)	0.0000
Chair Sit-and-Reach Test	Education—lower vs. higher level	−6.56 (−12.30, −0.81)	0.0258
	Occupational status—professionally inactive vs. professionally active	−6.63 (−10.24, −3.03)	0.0004
Back Scratch Test (right hand)	Occupational status—professionally inactive vs. professionally active	−6.94 (−11.75, −2.13)	0.0052
	WHtR	−31.85 (−63.40, −0.30)	0.0479
Back Scratch Test (left hand)	WC	−0.23 (−0.44, −0.01)	0.0388
8 Foot Up-and-Go Test	Age	0.17 (0.06, 0.28)	0.0034
	WC	0.06 (0.02, 0.11)	0.0095
	Time from diagnosis to study enrollment (years)	0.23 (0.11, 0.34)	0.0002

Abbreviations: 30 s Chair Stand Test, 30 s Arm Curl Test, 2 min Step-In-Place, Chair Sit-and-Reach Test, Back Scratch Test (right hand) Back Scratch Test (left hand) and 8 Foot Up-and-Go Test, specific tests included in the SFT; WC, waist circumference; WHR, waist-to-hip ratio; WHtR, waist-to-height ratio; 95% CI, 95% confidence interval.

## Data Availability

The data presented in this study are available on request from the corresponding author.

## Supplementary Materials

The following supporting information can be downloaded at: https://www.mdpi.com/article/10.3390/geriatrics9050125/s1, Table S1: Differences in SFT results by category of socio-demographic features with estimated effect sizes; Table S2: Basic characteristic of the SFT results according to the categories of obesity indices; Table S3: Differences in SFT results by categories obesity indicators with estimated effect sizes; Table S4: The SFT results (as reference categories) according to the categories of clinical and sociodemographic features and obesity indicators.
