# Supplementary material for: Influence of Obesity and Sociodemographic Features on the Physical Fitness of Breast Cancer Survivors
Source: Geriatrics (Basel). 2024 Oct 1;9(5):125. doi: 10.3390/geriatrics9050125 (PMC11507498; doi:10.3390/geriatrics9050125)
Supplement: Supplementary file 1 [file geriatrics-09-00125-s001.zip › geriatrics-3164740-supplementary.pdf]

Table 1. Differences in SFT results by category of socio-demographic features with estimated effect sizes.

Characteristic	30-second Chair Stand Test		30-second Arm Curl Test		2-minute Step-In-Place		Chair Sit-and-Reach Test		Back Scratch Test (right hand)		Back Scratch Test (left hand)		8 Foot Up-and-Go Test	
	Effect size (95% CI)	*P*	Effect size (95% CI)	*P*	Effect size (95% CI)	*P*	Effect size (95% CI)	*P*	Effect size (95% CI)	*P*	Effect size (95% CI)	*P*	Effect size (95% CI)	*P*
Age group	3.44	0.3281	0.56	0.9051	5.34	0.1484	1.08	0.7814	8.00	0.0461	7.57	0.0559	6.09	0.1072
Area of residence	-0.01 (-0.28, 0.27)	0.9575	-0.01 (-0.28, 0.26)	0.9422	-0.15 (-0.41, 0.13)	0.2912	-0.29 (-0.52, -0.01)	0.0450	-0.19 (-0.44, 0.09)	0.1863	-0.22 (-0.46, 0.06)	0.1288	-0.06 (-0.33, 0.22)	0.6661
Marital status	0.06 (-0.18, 0.30)	0.6187	0.04 (-0.20, 0.28)	0.7570	-0.05 (-0.29, 0.19)	0.6694	0.00 (-0.25, 0.24)	0.9728	-0.10 (-0.33, 0.15)	0.4413	-0.05 (-0.29, 0.20)	0.7035	-0.30 (-0.50, -0.06)	0.0153
Education	0.23 (-0.08, 0.50)	0.1585	0.06 (-0.25, 0.36)	0.7041	0.43 (0.14, 0.65)	0.0079	0.38 (0.08, 0.61)	0.0178	0.21 (-0.10, 0.48)	0.1938	0.20 (-0.11, 0.47)	0.2212	-0.03 (-0.33, 0.27)	0.8343
Occupational status	0.55 (0.04, 0.83)	0.0636	0.47 (-0.07, 0.80)	0.1125	0.40 (-0.16, 0.77)	0.1794	0.57 (0.07, 0.84)	0.0540	0.44 (-0.11, 0.78)	0.1433	0.19 (-0.37, 0.65)	0.5212	-0.83 (-0.94, -0.54)	0.0046
Comorbidities	-0.08 (-0.41, 0.27)	0.6639	0.20 (-0.15, 0.51)	0.2671	0.05 (-0.30, 0.38)	0.8078	0.07 (-0.28, 0.40)	0.7042	-0.02 (-0.35, 0.32)	0.9273	-0.16 (-0.48, 0.19)	0.3711	-0.08 (-0.41, 0.27)	0.6560

Abbreviations: 30-second Chair Stand Test, 30-second Arm Curl Test, 2-minute Step-In-Place, Chair Sit-and-Reach Test, Back Scratch Test (right hand), Back Scratch Test (left hand) and 8 Foot Up-and-Go Test, specific tests included in the Senior Fitness Test; 95% CI, 95% confidence interval.

Table 2. Basic characteristic of the SFT results according to the categories of obesity indices.

Characteristic			30-second Chair Stand Test	30-second Arm Curl Test	2-minute Step-In-Place	Chair Sit-and-Reach Test	Back Scratch Test (right hand)	Back Scratch Test (left hand)	8 Foot Up-and-Go Test
BMI									
	Normal weight	Mean (SD)	11.95 (4.20)	16.27 (4.14)	76.27 (25.74)	-3.23 (8.39)	-3.86 (10.25)	-9.86 (12.79)	9.23 (3.01)
		Median (IQR)	13.00 (4.00)	15.50 (6.00)	73.50 (41.00)	0.00 (8.00)	0.00 (18.00)	-6.50 (18.00)	8.50 (4.00)
		Min-Max	1.00-19.00	8.00-23.00	43.00-133.00	-24.00-8.00	-28.00-7.00	-38.00-4.00	6.00-18.00
	Overweight or obesity	Mean (SD)	11.77 (2.77)	15.62 (5.68)	81.12 (25.19)	-5.62 (9.38)	-10.03 (13.65)	-15.15 (13.37)	10.02 (4.24)
		Median (IQR)	12.00 (3.00)	15.50 (6.00)	78.00 (35.00)	-3.00 (11.00)	-9.00 (19.00)	-14.50 (13.00)	9.00 (2.00)
		Min-Max	5.00-20.00	3.00-46.00	26.00-127.00	-33.00-10.00	-64.00-23.00	-39.00-40.00	5.00-25.00
WC									
	Normal	Mean (SD)	12.65 (4.09)	16.91 (4.55)	82.39 (26.60)	-0.96 (7.75)	-2.65 (10.72)	-8.48 (12.19)	8.48 (3.10)
		Median (IQR)	13.00 (4.00)	16.00 (8.00)	78.00 (42.00)	0.00 (4.00)	2.00 (17.00)	-6.00 (17.00)	8.00 (3.00)
		Min-Max	1.00-20.00	8.00-24.00	43.00-133.00	-24.00-10.00	-28.00-7.00	-38.00-4.00	5.00-18.00
	Abdominal obesity	Mean (SD)	11.52 (2.74)	15.38 (5.55)	79.03 (24.94)	-6.46 (9.23)	-10.55 (13.32)	-15.72 (13.32)	10.29 (4.14)
		Median (IQR)	12.00 (3.00)	15.00 (6.00)	76.00 (34.00)	-4.00 (11.00)	-9.00 (18.00)	-15.00 (14.00)	9.00 (2.00)
		Min-Max	5.00-17.00	3.00-46.00	26.00-127.00	-33.00-6.00	-64.00-23.00	-39.00-40.00	5.00-25.00
WHR									
	Normal	Mean (SD)	14.40 (3.44)	19.20 (4.92)	89.00 (25.67)	-0.20 (4.60)	-0.40 (8.99)	-7.60 (4.28)	7.80 (2.68)

	Median (IQR)	13.00 (2.00)	21.00 (8.00)	84.00 (30.00)	0.00 (0.00)	4.00 (4.00)	-7.00 (8.00)	8.00 (2.00)
	Min-Max	11.00-20.00	13.00-24.00	61.00-126.00	-7.00-6.00	-16.00-6.00	-12.00--3.00	5.00-12.00
	Mean (SD)	11.66 (3.10)	15.58 (5.31)	79.36 (25.30)	-5.31 (9.30)	-8.98 (13.19)	-14.20 (13.63)	9.94 (4.01)
Abdominal obesity	Median (IQR)	12.00 (4.00)	15.00 (6.00)	76.00 (35.00)	-2.00 (11.00)	-7.00 (19.00)	-13.00 (16.00)	9.00 (2.00)
	Min-Max	1.00-19.00	3.00-46.00	26.00-133.00	-33.00-10.00	-64.00-23.00	-39.00-40.00	5.00-25.00
WHtR								
	Mean (SD)	14.71 (2.36)	19.57 (3.55)	96.57 (22.86)	1.57 (2.70)	3.00 (3.21)	-4.00 (6.43)	8.57 (2.82)
Normal	Median (IQR)	15.00 (3.00)	21.00 (8.00)	102.00 (38.00)	0.00 (5.00)	4.00 (6.00)	-4.00 (10.00)	8.00 (6.00)
	Min-Max	12.00-19.00	15.00-23.00	61.00-127.00	0.00-6.00	-2.00-7.00	-15.00-4.00	6.00-13.00
	Mean (SD)	11.57 (3.11)	15.46 (5.34)	78.47 (25.08)	-5.59 (9.30)	-9.48 (13.17)	-14.68 (13.48)	9.93 (4.04)
Obesity	Median (IQR)	12.00 (3.00)	15.00 (6.00)	76.00 (33.00)	-3.00 (10.00)	-9.00 (19.00)	-13.00 (15.00)	9.00 (2.00)
	Min-Max	1.00-20.00	3.00-46.00	26.00-133.00	-33.00-10.00	-64.00-23.00	-39.00-40.00	5.00-25.00

Abbreviations: 30-second Chair Stand Test, 30-second Arm Curl Test, 2-minute Step-In-Place, Chair Sit-and-Reach Test, Back Scratch Test (right hand), Back Scratch Test (left hand) and 8 Foot Up-and-Go Test, specific trials included in the SFT; BMI, body mass index; WC, waist circumference; WHR, waist-to-hip ratio; WHtR, waist-to-height ratio; SD, standard deviations; IQR, interquartile range; Min-Max, minimum-maximum.

Table 3. Differences in SFT results by categories obesity indicators with estimated effect sizes.

Characteristic	30-second Chair Stand Test		30-second Arm Curl Test		2-minute Step-In-Place		Chair Sit-and-Reach Test		Back Scratch Test (right hand)		Back Scratch Test (left hand)		8 Foot Up-and-Go Test	
	Effect size (95% CI)	*P*	Effect size (95% CI)	*P*	Effect size (95% CI)	*P*	Effect size (95% CI)	*P*	Effect size (95% CI)	*P*	Effect size (95% CI)	*P*	Effect size (95% CI)	*P*
BMI (kg/m^2)	0.13 (-0.15, 0.39)	0.3670	0.12 (-0.16, 0.38)	0.3977	-0.14 (-0.40, 0.14)	0.3399	0.14 (-0.14, 0.40)	0.3221	0.31 (0.04, 0.54)	0.0296	0.31 (0.04, 0.53)	0.0331	-0.12 (-0.38, 0.16)	0.4045
WC (cm)	-0.26 (-0.50, 0.01)	0.0630	-0.22 (-0.46, 0.05)	0.1183	-0.06 (-0.33, 0.21)	0.6484	-0.38 (-0.59, -0.12)	0.0073	-0.44 (-0.63, -0.19)	0.0018	-0.40 (-0.60, -0.14)	0.0048	0.39 (0.13, 0.59)	0.0055
WHR	-0.44 (-0.76, 0.05)	0.0987	-0.45 (-0.77, 0.04)	0.0910	-0.21 (-0.63, 0.30)	0.4329	-0.29 (-0.68, 0.22)	0.2800	-0.46 (-0.77, 0.03)	0.0899	-0.35 (-0.71, 0.16)	0.1940	0.42 (-0.08, 0.75)	0.1126
WHtR	0.60 (0.25, 0.82)	0.0079	0.57 (0.19, 0.80)	0.0133	0.43 (0.01, 0.72)	0.0609	0.47 (0.06, 0.74)	0.0388	0.67 (0.35, 0.85)	0.0034	0.57 (0.19, 0.80)	0.0133	-0.28 (-0.62, 0.16)	0.2239

Abbreviations: 30-second Chair Stand Test, 30-second Arm Curl Test, 2-minute Step-In-Place, Chair Sit-and-Reach Test, Back Scratch Test (right hand), Back Scratch Test (left hand) and 8 Foot Up-and-Go Test , specific tests included in the SFT; BMI, body mass index; WC, waist circumference; WHR, waist-to-hip ratio; WHtR, waist-to-height ratio; 95% CI, 95% confidence interval.

Table 4. The SFT results (as reference categories) according to the categories of clinical and sociodemographic features and obesity indicators.

Characteristic	Total	30-second Chair Stand Test		30-second Arm Curl Test		2-minute Step-In-Place		Chair Sit-and-Reach Test		Back Scratch Test (right hand)		Back Scratch Test (left hand)		8 Foot Up-and-Go Test	
		Below normal	Normal or above normal	Below normal	Normal or above normal	Below normal	Normal or above normal	Below normal	Normal or above normal	Below normal	Normal or above normal	Below normal	Normal or above normal	Below normal	Normal or above normal
Age group															
60-64 years	20 (22.7)	7 (30.4)	13 (20.0)	7 (36.8)	13 (18.8)	3 (8.6)	17 (32.1)	11 (24.4)	9 (20.9)	13 (26.0)	7 (18.4)	18 (26.1)	2 (10.5)	16 (21.1)	4 (33.3)
65-69 years	35 (39.8)	8 (34.8)	27 (41.5)	6 (31.6)	29 (42.0)	17 (48.6)	18 (34.0)	16 (35.6)	19 (44.2)	16 (32.0)	19 (50.0)	24 (34.8)	11 (57.9)	34 (44.7)	1 (8.3)
70-74 years	14 (15.9)	2 (8.7)	12 (18.5)	4 (21.1)	10 (14.5)	5 (14.3)	9 (17.0)	7 (15.6)	7 (16.3)	10 (20.0)	4 (10.5)	10 (14.5)	4 (21.1)	10 (13.2)	4 (33.3)
75 or older	19 (21.6)	6 (26.1)	13 (20.0)	2 (10.5)	17 (24.6)	10 (28.6)	9 (17.0)	11 (24.4)	8 (18.6)	11 (22.0)	8 (21.1)	17 (24.6)	2 (10.5)	16 (21.1)	3 (25.0)
Side															
Both side	11 (12.5)	4 (17.4)	7 (10.8)	3 (15.8)	8 (11.6)	5 (14.3)	6 (11.3)	6 (13.3)	5 (11.6)	7 (14.0)	4 (10.5)	10 (14.5)	1 (5.3)	10 (13.2)	1 (8.3)
Left side	47 (53.4)	12 (52.2)	35 (53.9)	10 (52.6)	37 (53.6)	18 (51.4)	29 (54.7)	24 (53.3)	23 (53.5)	26 (52.0)	21 (55.3)	34 (49.3)	13 (68.4)	40 (52.6)	7 (58.3)
Right side	30 (34.1)	7 (30.4)	23 (35.4)	6 (31.6)	24 (34.8)	12 (34.3)	18 (34.0)	15 (33.3)	15 (34.9)	17 (34.0)	13 (34.2)	25 (36.2)	5 (26.3)	26 (34.2)	4 (33.3)
Lymhadenectomy															
No	56 (63.6)	13 (56.5)	43 (66.2)	7 (36.8)**	49 (71.0)**	20 (57.1)	36 (67.9)	25 (55.6)	31 (72.1)	31 (62.0)	25 (65.8)	45 (65.2)	11 (57.9)	47 (61.8)	9 (75.0)
Yes	32 (36.4)	10 (43.5)	22 (33.9)	12 (63.2)**	20 (29.0)**	15 (42.9)	17 (32.1)	20 (44.4)	12 (27.9)	19 (38.0)	13 (34.2)	24 (34.8)	8 (42.1)	29 (38.2)	3 (25.0)
RTH															
No	46 (52.3)	12 (52.2)	34 (52.3)	13 (68.4)	33 (47.8)	18 (51.4)	28 (52.8)	26 (57.8)	20 (46.5)	27 (54.0)	19 (50.0)	38 (55.1)	8 (42.1)	39 (51.3)	7 (58.3)

Yes	42 (47.7)	11 (47.8)	31 (47.7)	6 (31.6)	36 (52.2)	17 (48.6)	25 (47.2)	19 (42.2)	23 (53.5)	23 (46.0)	19 (50.0)	31 (44.9)	11 (57.9)	37 (48.7)	5 (41.7)
CHTH															
No	44 (50.0)	14 (60.9)	30 (46.2)	12 (63.2)	32 (46.4)	19 (54.3)	25 (47.2)	26 (57.8)	18 (41.9)	23 (46.0)	21 (55.3)	34 (49.3)	10 (52.6)	41 (54.0)	3 (25.0)
Yes	44 (50.0)	9 (39.1)	35 (53.9)	7 (36.8)	37 (53.6)	16 (45.7)	28 (52.8)	19 (42.2)	25 (58.1)	27 (54.0)	17 (44.7)	35 (50.7)	9 (47.4)	35 (46.1)	9 (75.0)
Domicile															
Rural	22 (25.0)	8 (34.8)	14 (21.5)	6 (31.6)	16 (23.2)	11 (31.4)	11 (20.8)	15 (33.3)	7 (16.3)	16 (32.0)	6 (15.8)	20 (29.0)	2 (10.5)	19 (25.0)	3 (25.0)
Urban	66 (75.0)	15 (65.2)	51 (78.5)	13 (68.4)	53 (76.8)	24 (68.6)	42 (79.3)	30 (66.7)	36 (83.7)	34 (68.0)	32 (84.2)	49 (71.0)	17 (89.5)	57 (75.0)	9 (75.0)
Marital status															
In a relationship	51 (58.0)	14 (60.9)	37 (56.9)	10 (52.6)	41 (59.4)	22 (62.9)	29 (54.7)	26 (57.8)	25 (58.1)	31 (62.0)	20 (52.6)	39 (56.5)	12 (63.2)	42 (55.3)	9 (75.0)
Single	37 (42.1)	9 (39.1)	28 (43.1)	9 (47.4)	28 (40.6)	13 (37.1)	24 (45.3)	19 (42.2)	18 (41.9)	19 (38.0)	18 (47.4)	30 (43.5)	7 (36.8)	34 (44.7)	3 (25.0)
Education															
Higher level	72 (81.8)	15 (65.2)*	57 (87.7)*	14 (73.7)	58 (84.1)	24 (68.6)**	48 (90.6)**	34 (75.6)	38 (88.4)	37 (74.0)*	35 (92.1)*	56 (81.2)	16 (84.2)	61 (80.3)	11 (91.7)
Lower level	16 (18.2)	8 (34.8)*	8 (12.3)*	5 (26.3)	11 (15.9)	11 (31.4)**	5 (9.4)**	11 (24.4)	5 (11.6)	13 (26.0)*	3 (7.9)*	13 (18.8)	3 (15.8)	15 (19.7)	1 (8.3)
Occupational status															
Professionally active	4 (4.6)	0 (0)	4 (6.2)	0 (0)	4 (5.8)	0 (0)	4 (7.6)	0 (0)*	4 (9.3)*	0 (0)*	4 (10.5)*	2 (2.9)	2 (10.5)	1 (1.3)***	3 (25.0)***
Professionally inactive	84 (95.5)	23 (100.0)	61 (93.9)	19 (100.0)	65 (94.2)	35 (100.0)	49 (92.5)	45 (100.0)*	39 (90.7)*	50 (100.0)*	34 (89.5)*	67 (97.1)	17 (89.5)	75 (98.7)***	9 (75.0)***
Comorbidities															
No	12 (13.6)	3 (13.0)	9 (13.9)	1 (5.3)	11 (15.9)	4 (11.4)	8 (15.1)	5 (11.1)	7 (16.3)	7 (14.0)	5 (13.2)	11 (15.9)	1 (5.3)	10 (13.2)	2 (16.7)
Yes	76 (86.4)	20 (87.0)	56 (86.2)	18 (94.7)	58 (84.1)	31 (88.6)	45 (84.9)	40 (88.9)	36 (83.7)	43 (86.0)	33 (86.8)	58 (84.1)	18 (94.7)	66 (86.8)	10 (83.3)
BMI group															
Normal weight	22 (25.0)	6 (26.1)	16 (24.6)	3 (15.8)	19 (27.5)	11 (31.4)	11 (20.8)	8 (17.8)	14 (32.6)	8 (16.0)*	14 (36.8)*	13 (18.8)**	9 (47.4)**	18 (23.7)	4 (33.3)
Overweight or obesity	66 (75.0)	17 (73.9)	49 (75.4)	16 (84.2)	50 (72.5)	24 (68.6)	42 (79.3)	37 (82.2)	29 (67.4)	42 (84.0)*	24 (63.2)*	56 (81.2)**	10 (52.6)**	58 (76.3)	8 (66.7)
WC group															
Normal	23 (26.1)	6 (26.1)	17 (26.2)	2 (10.5)	21 (30.4)	8 (22.9)	15 (28.3)	5 (11.1)**	18 (41.9)**	6 (12.0)***	17 (44.7)***	12 (17.4)***	11 (57.9)***	16 (21.1)**	7 (58.3)**
Abdominal obesity	65 (73.9)	17 (73.9)	48 (73.9)	17 (89.5)	48 (69.6)	27 (77.1)	38 (71.7)	40 (88.9)**	25 (58.1)**	44 (88.0)***	21 (55.3)***	57 (82.6)***	8 (42.1)***	60 (79.0)**	5 (41.7)**
WHR group															
Normal	5 (5.7)	1 (4.4)	4 (6.2)	0 (0)	5 (7.3)	2 (5.7)	3 (5.7)	1 (2.2)	4 (9.3)	1 (2.0)	4 (10.5)	3 (4.4)	2 (10.5)	3 (4.0)	2 (16.7)
Abdominal obesity	83 (94.3)	22 (95.7)	61 (93.9)	19 (100.0)	64 (92.8)	33 (94.3)	50 (94.3)	44 (97.8)	39 (90.7)	49 (98.0)	34 (89.5)	66 (95.7)	17 (89.5)	73 (96.1)	10 (83.3)
WHtR															
Normal	7 (7.9)	0 (0)	7 (10.8)	0 (0)	7 (10.1)	2 (5.7)	5 (9.4)	0 (0)**	7 (16.3)**	0 (0)**	7 (18.4)**	3 (4.4)*	4 (21.1)*	5 (6.6)	2 (16.7)
Obesity	81 (92.1)	23 (100.0)	58 (89.2)	19 (100.0)	62 (89.9)	33 (94.3)	48 (90.6)	45 (100.0)**	36 (83.7)**	50 (100.0)**	31 (81.6)**	66 (95.7)*	15 (79.0)*	71 (93.4)	10 (83.3)

Note: Data are presented as number (percentage). Abbreviations: 30-second Chair Stand Test, 30-second Arm Curl Test, 2-minute Step-In-Place, Chair Sit-and-Reach Test, Back Scratch Test (right hand), Back Scratch Test (left hand) and 8 Foot Up-and-Go Test , specific tests included in the Senior Fitness Test; RTH, radiotherapy; CHTH, chemotherapy; BMI, body mass index; WC, waist circumference; WHR, waist-to-hip ratio; WHtR, waist-to-height ratio; *, $P<0.05$; **, $P<0.01$; ***, $P<0.001$.
